# Post-Traumatic Stress Disorder and Urban Violence: An Anthropological Study

**DOI:** 10.3390/ijerph10115333

**Published:** 2013-10-25

**Authors:** Juliana Da Silva-Mannel, Sérgio Baxter Andreoli, Denise Martin

**Affiliations:** Federal University of São Paulo, Rua Borges Lagoa, 570, Vila Clementino, São Paulo, SP 04038-020, Brazil; E-Mails: andreoli@dpsiq.epm.br (S.B.A.); denise.martin@unifesp.br (D.M.)

**Keywords:** urban violence, post-traumatic stress disorder (PTSD), qualitative research, anthropology

## Abstract

The study aimed to understand how “distress” is experienced by patients with Post-Traumatic Stress Disorder (PTSD) in the social-cultural context of São Paulo, Brazil, an urban environment marked by social inequality and high levels of violence. A qualitative study was conducted between 2008 and 2010 with PTSD patients (F43.1, ICD-10, 1997) who had been victims of robberies and kidnappings in São Paulo. Dense ethnographic observations were carried out, as well as in-depth semi-structured interviews with ten adult patients. The analysis method used was based on anthropology. The results show that it is particularly important to distinguish between perceptions of different forms of the experience of social suffering and perceptions of health and illness held by victims and biomedical experts. The cause of PTSD is more often associated with the personal problems of the victim than with the specific traumatic event. The distress described in terms of what is considered a “normal” reaction to violence and what is considered a symptom of PTSD. The findings indicate that the diagnostic of PTSD can be understood in relation to the different contexts within a culture. The ethnographic approach serves not only to illuminate individual suffering but also the social suffering experienced by the residents of São Paulo.

## 1. Introduction

Post-Traumatic Stress Disorder (PTSD) is a recent diagnostic category that was established in 1982 in the third edition of the Diagnostic and Statistical Manual of Mental Disorders (DSM-III). The first studies on PTSD were focused on the psychological distress of ex-combatants of the Vietnam War; here, the traumatic event was considered extraordinary, an event occurring outside the usual routine. After the definition of “traumatic event” was expanded to include any situation that involves a loss of “physical integrity” or the risk of death or serious injury, in the 1994 edition of the DSM-IV, an increase of new cases was noted in the population in general [1].

PTSD has become the concept most often used to explain the psychiatric consequences caused by different situations of exposure to violence [2]. The main risk factors for development of PTSD after a traumatic event are considered to be the following: demographic variety, war, post-war and other violent environments, poverty, and social inequality [3]. Urban violence has also been suggested as a risk factor specific to the populations of large cities [4,5]. A study realized in two large Brazilian cities showed that 63.06% of the participants in Rio de Janeiro and 59.4% in São Paulo faced lifetime traumatic events related to assaultive violence, and among those 8.7% in Rio de Janeiro and 10.2% in Sao Paulo suffered PTSD [6].

The scholarly literature about PTSD is extensive. Different studies have sought to clarify the roots of PTSD as neuroscience and psychiatry data suggest biological, neurobiological or even genetic risk factors for PTSD [3,7,8]. Most of the epidemiological studies have showed specific traumatic events in specific populations, such as intimate partner violence among women, domestic violence among chidren, or traumatic events in conflict and post-conflict settings [5,9,10,11,12]. The revision of the literature about PTSD showed that the most of studies have focused primarily on epidemiology and this is the first qualitative study about PTSD conducted in Brazil.

Some researchers have questioned the universal diagnosis of PTSD, mainly because it is considered to be fairly limited as a diagnostic category when applied across different populations and cultures [13,14,15,16,17,18]. Critics say that there is a multiplicity of different societies and cultures in the world where collective violence influences emotions and reactions in different ways and suffering is interpreted differently, without necessarily matching any specific definitions of disorders or diseases. Furthermore, such critics claim that PTSD is a diagnostic category that was created, rather than discovered, based on socio-political needs. According to the World Report on Violence and Health [19], “collective violence is the instrumental use of violence by people who identify themselves as members of a group against another group or set of individuals, in order to achieve political, economic or social objectives” ([19], p.5). Examples are acts of terrorism, organized violent crimes, wars, genocide, repression and other human rights abuses.

Since the 70s there has been an accelerating increase in all violent criminal modalities throughout Brazil, particularly in such modalities related to tensions in inter-subjective relationships, not related directly to crime, but related to conflicts between people passing in public places, family members, neighbours, employers and employees, and so on [20].

The rate of homicides in Brazil is one of the highest in the World. In the 15 years between 1992 and 2007, the homicide rate grew 32%, from 19.2 deaths per 100,000 inhabitants to 25.4 [21]. The figure has been gradually coming down since 2003, when a record of 28.8 deaths per 100,000 inhabitants was reached [22]. According to available statistics, homicides are often concentrated in metropolitan areas, in particular among young males. In fact, 59.8% of all homicides among young people in Brazil in 2003 occurred within the metropolitan regions of São Paulo and Rio de Janeiro [23]. These data correspond to the period in which this research was being carried out.

The latest research on homicides in Brazil were presented in March 2013 through the document named “Mapa da violência 2013”, which refers to sources of the Ministry of Health in Brazil and the World Health Organization [19]. The data showed a rate of 20.4 homicides per 100,000 inhabitants, the eighth worst mark in a ranking with 100 other countries. According to this document, in 2010 36,792 people were murdered, surpassing the year 2009 which recorded 36,624 deaths from firearms. This document showed that violent deaths were concentrated mostly in the large cities tend to spread across the country, because of industrial de-concentration and population displacements linked to economic activities.

Cardia *et al*. [24] have discussed why homicides are often concentrated in urban areas, although not all such areas have high homicide rates. They claim that violence is generally most intense where social and economic rights are limited. They also emphasize that the violence in Brazil is not the result of revolution, civil war or theocratic government, but is rather a continuation of long-standing problems, a consequence of the transition from authoritarian rule to democracy.

Particularly in São Paulo, in a context marked by violence and high levels of social inequality [25], urban space has undergone huge transformation resulting in the formation of new forms of organization of social life (monitored and closed commercial complexes, monitored condominiums of apartments and houses, shopping malls, *etc*.). This process includes changes in the specific terms of social relationships between the inhabitants (restriction of people’s access to each other, creation of stereotypes, proliferation of social discrimination, *etc*.) because of exacerbated feelings of fear and insecurity [26]. According to Martin *et al*. [27], such social changes might influence the subjectivity of individuals, and these changes might be expressed in various forms of suffering and disturbances.

The purpose of this article is to discuss the “distress” experienced by victims of urban violence diagnosed with PTSD. The anthropological approach adopted in the study treats PTSD in relation to the cultural context of the sufferer. Different levels of specific understanding are therefore considered within populations where the suffering is identified and legitimized. The anthropological basis means that the analysis contributes to psychiatry and public health debates surrounding the understanding of the relationship between PTSD and problems caused by urban violence. The paper is also intended to contribute to the establishing of more culturally appropriate interventions and healing strategies for the population studied.

## 2. Methodology

A qualitative study was conducted between October 2008 and March 2010 among people who had been victims of assaults and kidnappings in the Brazilian city of São Paulo in Brazil, and who had been diagnosed with PTSD (F43.1, ICD-10, 1997). The selection of the participants of the research took place in connection with the “Programa de Atendimento e Pesquisa em Violência” (PROVE). This is a psychiatric care service specializing in the treatment of victims of violence, which is further linked to a research programme on violence at the Department of Psychiatry at the Federal University of São Paulo.

São Paulo is the largest city in Brazil, with an urban population of 11 million people and a total population of 19 million including the metropolitan region [28]. The city has been considered the country’s most important centre of industry, commerce and finance, but statistical data show that 10% of the population lives below the poverty line [22]. The wealthy classes of the population are concentrated in the central regions of the city, while much of the poverty concentrates in the periphery, forming a belt of areas where infrastructure is almost non-existent, socioeconomic conditions are poor, and serious violations of human rights take place [25]. The rate of homicides in São Paulo grew from 38.92 cases per 100,000 inhabitants in 1993 to 47.63 in 2003 [29]. The figure had been significantly reduced to 15.0 by 2007, but the incidence of violent crime is still very high, in particular among young males living in the poorest areas [21].

**Table 1:** General Description of Interviewees. São Paulo/SP, 2008–2010.

Name	Sex	Age	Marital Status	Educational Stage	Religion	Occupation	Traumatic Event
ED	M	35	Married	Secondary School	Catholic	Bus driver	Robbery
EL	F	31	Single	Bachelor	Spiritist	Journalist	Kidnapping
G	M	28	Married	Secondary School	Any	Policeman	Kidnapping
JC	M	25	Married	Secondary School	Spiritist	Corporate Auxiliary	Robbery
JO	M	46	Married	Primary School	Spiritist	Caretaker	Kidnapping
L	F	44	Married	Bachelor	Catholic	Physiotherapist	Robbery
M	F	33	Single	University Student	Catholic	University Student	Robbery
PA	M	39	Married	Secondary School	Catholic	Policeman	Kidnapping
PT	F	32	Separate	Secondary School	Spiritist	Policewoman	Kidnapping
S	F	31	Cohabiting	Secondary School	Catholic	Seller	Robbery

**Table 2:** A brief description of traumatic events: robberies and kidnappings. São Paulo/SP, 2008–2010.

After working the night shift from Saturday to Sunday **ED** stopped on his way home to buy some fried chicken for the family dinner. Entering the shop he soon became a victim of a robbery that occurred there. He was used as a protective shield by the gunman in a shooting with the shop owner. There were no casualties.
**EL** had been involved in the negotiations to rescue her boyfriend, who was kidnapped for 33 days. She began to feel different a few months after the kidnapping. She realized that her symptoms became worse during her work as a screenwriter of a television programme that treated violence in a sensational way.
**G** took a lift home with two work colleagues when a motorcycle stopped beside the car and announced a robbery. When the robbers saw the person in police uniform in the back seat of the car the robbery turned into a kidnapping. The victims were held by the kidnappers for more than five hours. There was a lot of physical violence and shooting.
**JC** was assaulted twice and suffered three attempted assaults during the time he spent working as an administrative assistant at an engineering firm. He carried out deposits and bank drafts of high value for the company. He began to feel strange after the last robbery, in which he handed the envelope with the money to the robber but the robber did not believe the money was inside, and threatened to kill him.
**JO** was kidnapped in his own car in front of his house and spent five hours with the kidnappers. He was tortured because he did not have any cards with him and was unable to withdraw money from any cash machine. Finally he was abandoned in a slum, in his own car but without the keys. When he went to get help his car was stolen. He did not leave home for five years, working during that time as a janitor in the same building in which he lived.
**L** was in her car on her way to work when she was forced to stop by another car that was following her. Three men entered her car and forced her to drive back home. She and her husband were in the hands of the criminals for three and a half hours. They stole things from their home and also took her car. She learned at the police station that the same gang had already assaulted other residents in the neighbourhood.
**M** was mugged at night not far from her home after getting off the bus from college. Two men on a motorbike pulled on her backpack so that she fell to the ground. She suffered minor injuries.
**PA** was driving home after work with two colleagues. At a traffic light, two men on a motorbike stopped beside the car and announced an assault. When the criminals saw that the passenger on the back seat was wearing a police uniform the assault turned into a kidnapping. There was a lot of physical violence and an exchange of fire.
**PT** was returning from work with two colleagues. She was sitting on the back seat when she saw two people on a motorcycle pointing a gun at the driver, announcing a robbery. When the robbers saw her, she was identified as a policewoman because of her uniform. The two robbers entered the car and forced the driver to drive to a slum. PT was made to watch her colleagues being beaten up before she was also beaten.
**S** was mugged four times over the course of three years, twice on her way back from work, once inside her house and once at the shop where she worked. After the last assault, when she returned from work she would feel sad, discouraged and aggressive, and cried easily. After she was dismissed from her job these symptoms got worse.

To be included in this study, participants were required to live in São Paulo. Ten patients were selected, five men and five women, aged 25 to 47 years (average 34.7 years). The participants were chosen because they were the most articulate and informative and met the following inclusion criteria: (1) adult with a diagnosis of PTSD; (2) victim of assault and/or kidnapping; (3) showing evidence of serious mental suffering (disturbance) after having lived through one of these events; (4) under psychiatric treatment for a period greater than three months. See Table 1 for a description of the participants selected and Table 2 for a brief description of traumatic events.

The fieldwork took place in the PROVE office and in the domiciles of the participants. The participants’ names were replaced with initials. Dense ethnographic observations and in-depth semi-structured interviews were conducted until saturation of the guideline content was reached [30,31]. The data were recorded and transcribed. The analysis consisted of reading the transcriptions and grouping the content categories. The results were interpreted using a theoretical anthropological approach to cultural concepts [32,33]. Acts, events, words and interpretations were used to form a logical model to explain the real-life contexts of the participants of the research. 

The study was approved by the ethics committee at UNIFESP (procedural No. 1356/07); all participants were informed about its objectives and provided their written consent.

## 3. Results and Discussion

### 3.1. The Traumatic Event: What Happened to Me

The interviewees mentioned that the occurrence of violence in São Paulo was not a novelty for them. They knew that some areas in the city were more violent than others, and that some places were considered more dangerous in terms of high incidence of robberies, thefts, drug trafficking and homicides. However, they did not believe that they would actually become victims of violence. They believed violence happened to “other people” in different geographical or social categories. The interviewees considered themselves to be workers, *i.e*., people whose status between middle class and poverty would make them unlikely to become targeted by crimes such as robbery and kidnapping. The following statements illustrate this attitude.

“I was attacked when I was on my way to work at ten to seven in the morning. I worked for 15 years at the same place and I had also been working the night before. Nothing had ever happened to me there, although we knew some people used to get killed in the area. It happened just when I was driving around the weir; he was already behind me and I had not seen him before... Then he stopped his car in front of mine; he made me get out of my car and then they [the other members of the gang] stayed with me in the car (...) It was terrible! The breathlessness that I had when I stayed with them in the car, wow… I said this: I’ll faint! Then they said: If you faint, you’ll die! (…) He asked: You have a plasma TV? I said: No, I’m a worker. So he asked me why I had no tinted glass on my car to protect me. They stayed about three hours in my home… Do I say ‘thank God that everything worked out well in the end’? No, I don’t. I would say thank God if nothing like this had happened. Considering what has happened, was I lucky? Yes, because I talked too much... I could have got shot, because he ordered me to shut up many times. So, that’s all... Today, if you do anything, you’ll get shot... It is something that bothers me.” (L, 44 years, physiotherapist)*.*

“(…) It was on this street where I was attacked. They came out from behind a tree here at the end of my street and approached me in the middle of the hill... It could have happened to anyone at that time, you know! It was the right time for those guys. They picked the right time for them but for me it was the wrong time. I think that this is what happened. I think that unfortunately this happens all the time; it is poor people, mugging poor, you know. It is more or less like this... As you can see, I have a simple house, I have no car (...) I had not a cent in my pocket. I have no credit card and no chequebook and I usually walk without documents. You see, I had not even lost my documents. They took nothing from me, just my peace, you know! This is something that has no structure. São Paulo has everything but the structure to deal with this violence. Too many people! People in need of education, people in need of work and then they cause these things, you know! And now, as the guy has no structure, he will mug other people! It’s a chain reaction... And they are certainly people from the region... I didn’t see [if the men were from the region], because I stayed inert. I didn’t even move. I was afraid that they could drag me by my backpack, because they rode a motorcycle and they were two” (M, 31 years, university student).

The narratives of the interviewees not only show how people of a particular social group—workers —may become victims of robbery and kidnapping in São Paulo, but also, primarily, demonstrate the unequal distribution of social and civil rights in Brazilian society. As shown in the above statements, the interviewees feel that the problem of violence in São Paulo is a kind of “fight” justified by income inequality and lack of social security. However, this justification operates on an individual rather than a collective level, *i.e*., their criticism is neither directed at the State nor at the (dis)organization of society. The interviewees tend to reduce the problem to the direct relationship between victim and culprit, between one person and another, associating the violence with the causal relationship between poverty and crime. In other words, according to this understanding, the poor commit crimes because of their poverty.

In her study on violence in the poor areas of Rio de Janeiro in the 1980s, Zaluar [34] illustrates how the causal relationship between poverty and crime is pejorative and can be perverse, because it leads to discrimination against the poor, both in the people’s imaginations and in the institutions responsible for suppressing the behaviour considered criminal. Poverty alone is not a factor that justifies violence, but the most serious human rights violations happen in areas with a high level of poverty. This relationship between violation of human rights and poverty (social and economic lakes) constitutes a fertile field for violence to thrive.

According to Cardia *et al*. [24] the problem of violence in urban areas in Brazil cannot be properly understood and prevented without recognizing that large sectors of those urban populations have very little access to many social and economic rights. They argue that serious human rights violations usually accompany profound social inequalities and/or authoritarian regime—such as the Brazilian dictatorship, which only ended in 1985. They also observe that, concurrent with the change in the political regime, the rate of violent crimes has increased. Further, sharp changes in economic policies have led to an increase in inequalities. Despite the reintroduction of democracy to Brazil, serious human rights violations perpetrated by the police or organized groups such as death squads are regularly documented by the media.

“Death by banal reason” was an expression used by the interviewees to refer to their fragile state during the violent event. The fear of dying during a robbery or kidnapping was described as an intense emotion. The interviewees felt vulnerable to the possibility of being executed at any time and felt there was no chance of dialogue during the confrontation. They described their reactions in the moments where they felt dominated by the criminals by referring to breathlessness, fainting, tremors, body paralysis, inertia, and tachycardia. The emotions described by the interviewees included fear, anger, shame, guilt, humiliation and insecurity.

According to Adorno [20], the exacerbation of feelings of fear and insecurity among the population has provoked deep impacts on social, cultural, economic and political life throughout Brazil. Adorno suggests that the measures taken by the population to protect themselves because of fear and insecurity tend to contribute to the further weakening of the solutions provided by the law and the criminal justice system. Caldeira [26] shows how the fear of becoming a victim of violence has become a justification for the creation of a number of private protection strategies that have helped generate a new pattern of urban segregation, such as the construction of privatized, closed and monitored spaces for residence, leisure, work and consumption. Caldeira suggests that these “walls” act both symbolically and materially, because they “create differences, impose divisions and distances, lead to separations, multiply rules of avoidance and exclusion” ([26], p. 9).

### 3.2. Stress of Violence: The Request for Help and the Identification of PTSD

The interviewees sought help in different ways and over very different time periods (from one month to five years after the event). They found the care service specializing in PTSD through the municipal health network, electronic media or articles in magazines, or on television. All of them obtained treatment primarily because they had been victims of violence. The reason most of them gave for their delay in turning to the care service was that they had not thought they could fall ill because of the assault and/or the kidnapping. They also mentioned that they had not had any notion of PTSD or traumatic distress, regarding their problem only as biological, *i.e*., a matter of physical pain. Furthermore, some of the interviewees did not believe in the effectiveness of psychological and psychiatric treatment.

The interviewees in many cases tried to forget what had happened to them and avoided any remembrance of the violent event. In describing their suffering after the event the interviewees mentioned intense fear, breathlessness, headaches, sadness, an inability to sleep, nightmares, a constant state of alert, and palpitations (tachycardia). These reactions became more intense in situations with unknown people, for example in the tube, in traffic or at work. The expressions “emotional side” and “the thrill of the body” were mentioned as explanations for the “abnormal state”. This attitude is illustrated in the following comments.

“I think I’m treating that side, the psychological side. Something that fell apart inside my body, emotional, you know? (…) this is what I feel and if there was any chemical reaction that makes you stay quiet, calm, which is not happening in my case, I think she (doctor) will apply medicine because it was after that (assault), that everything got worse, understand? Let’s say it like this: you were a person impatient by nature? It got worse. You were a person who often suffers from headaches? It got worse… understand? Everything got worse because headaches, migraines, I’ve always had... since childhood, but it wasn’t a constant thing, and after the assault it got worse, every day, no lie.” (M, 31 years, university student).

“I still don’t know if it’s a disease or an emotion of the body itself, or of the character… I don’t know; I can’t tell if it’s normal or abnormal (...) What do you mean with treatment? ... ah, this insomnia, you know? Even the psychologist... Ah... this type of treatment... this motivation (feeling) of anger, of bitterness, of all this. I was almost a month without sleep, all the time remembering, with that feeling of anger and hate, revenge as well. So you need to get treated so you don’t end up doing bullshit.” (G, 28 years, policeman)

The interviewees’ description of their suffering did not appear linked to their conception of PTSD. This distinction of explanatory models—biomedical and non-biomedical—shows that there are different understandings of the impact of urban violence on mental health. It might be argued that the victims of urban violence use various forms to express their anguish and pain in facing up to the violent event experienced, when all biomedical categories fail.

According to Duarte [35], “disease”, denoting a certain kind of suffering and disturbance, is a category directly linked to the biomedical way of thinking, which in fact is only one of many ways of understanding the world and human life. Herzlich [36] emphasizes that biomedicine produces social categories of health and disease, but that different social groups have diverse conceptions of the etiology of diseases, responding to a logic that is different to that used in medicine. In the same study, Herzlich explains that to interpret phenomena of the body, people rely on concepts, symbols and internalized reference schemes according to their culture. A number of studies have contributed to research into the innumerable expressions and manifestations of unspecific complaints and somatic pains in different cultural groups. Such authors include Kleinman [37,38,39], Littlewood [40,41], Good [42,43], Kirmayer [44,45] and Laplantine [46].

According to Kienzler [13], most studies on the impact of violence on mental health are based on theories specifically relating to war trauma, PTSD and other trauma-related disorders. This approach has led to the development of different programmes and care services to help victims of violence; usually, however, the Western biomedical model, *i.e*., based on traditional psychosocial, pharmacological and cognitive-behavioural interventions, is still applied.

There are two distinct standpoints in the debate about PTSD. On the one hand, some clinicians and epidemiologists attempt to define PTSD as a universal, cross-culturally valid psychopathological response to traumatic distress, which may be cured or ameliorated by clinical and psychosocial therapeutic measures [3,47,48]. On the other hand, transcultural psychiatrists and anthropologists tend to maintain that the discourse of post-traumatic stress disorder can only be valid in a Western cultural and moral context [49,50]. These commentators hold that importing the concept of PTSD and associated treatment can have a destabilizing effect on cultural-specific forms of post-conflict and post-war organization, by providing “rapid therapeutic solutions” that confuse people in their attempts to reconstruct relations after extreme experiences [2]. Some authors [15,16,51] have provided evidence to reinforce the hypothesis that every cultural or social group develops its own symptoms and mental disorders in response to violence; what is normal and what is not is distinguished by diverse forms of experience and behaviour, as judged by the members of a particular group. The question is whether suffering can be alleviated within a social and familial environment, or through socio-cultural, economic and religious activities which together define the symbolic cultural universe of the person concerned, making the world understandable before, during and after the conflict. In addition, Young [18] and Summerfield [52] have emphasized that PTSD was a diagnosis mainly invented to satisfy socio-political needs and respond to the interests of psychiatric institutions and ex-combatants of the Vietnam War, rather than a psychopathological phenomenon subjected to proper investigation.

Although the biomedical and anthropological standpoints appear mutually exclusive, on both sides the debate has led to the development of less radical approaches. PTSD is a mental disorder precipitated by an external cause, and therefore cannot be understood exclusively as “internal”. To understand PTSD, external factors need to be taken into account, which inevitably involves the social-cultural context. The only exception is trauma caused by natural disasters.

One specific contribution of anthropology to the discussion on PTSD is the concept of *social suffering* [14,53,54,55]. This relates to the idea that although violence can lead to a very personal and subjective experience of pain, the influence of social-macro actors—State, international organizations, global media, cross-border financial flows—needs to be taken into account. These actors can facilitate, maintain or combat violence. The concept of social suffering involves understanding pain and trauma without focusing only on the individual dimension. Suffering is considered not as a medical or psychological problem, but as a social experience that brings together different experiences of pain, different situations of violence and oppression, and different conceptions of morality, health, justice and religion [56].

### 3.3. The Relationship between PTSD and Urban Violence: The Individualist Point of View

The interviewees did not recognize urban violence as a cause of PTSD. Although they were diagnosed with PTSD because of the experience of assault and/or kidnapping, crimes related to urban violence, the interviewees believed their personal life was the cause for their distress. Their narratives have some distinct characteristics in common: the justification of PTSD as an accumulation of traumas experienced throughout life; evidence of personal fragility, a feeling of not being able to endure life’s problems or the difficulties imposed by the city of São Paulo; the sense of divine punishment as a way of learning to become a better person. The following quotes illustrate these points:

“So the truth is, I don’t know what the doctor told you, but my life was not just a kidnapping that happened. I think that several points in my life… problems I had when I was eight I have until today, insecurity, these symptoms (...) do not know if all these traumas were joining until they accumulated (to PTSD).” (EL, 31 years, journalist).

“Since then, since my childhood, what my mother told my dad, you know, my father drank, had other women, talking like that, now in the family. And me, being the oldest son, I saw all this, I grew up in the middle of trickery and drugs (...) and every day that goes by, is a part of the war that we won, because what we see nowadays in the TV news is absurd.” (ED, 35 years, bus driver).

“I’m Catholic but not practising. When it started (referring to symptoms of PTSD), I began to ask why I wasn’t going (to church). Then I started to frequent a spiritual centre to meet and stuff, and ... I started to realize that ... Hmm ... from a spiritual view on that here, you are like a kind of apprentice, so it was where I started, because if I was…(assaulted), if I had (PTSD)… that is because somehow... before incarnating here on this spiritual level, I was destined to go through certain types of situations, so I could learn and appreciate certain things, so I made up my mind, and saw that it did not happen by accident. Actually, my separation with my wife also did not happen by accident, maybe I did not give too much value to her, our marriage is ... after I ... we began to live together, then we broke up, right, so I guess nothing happened by chance, I think I needed to separate to give more value to her, and ... I don’t know, suddenly I had to go through that assault to be able to wake up in the company, trying to change something.” (JC, 25 years, corporate assistant).

The interviewees added other personal experiences of suffering, trying to find a logical response to their PTSD. From the stories they narrate emerges a meaning permeated by the notion of “the individual”. It can be argued that this is included in the narratives as an attempt to give meaning to the diagnosis of PTSD; this would seem to validate PTSD as a universal, cross-cultural diagnostic category. For those interviewed, the notion of “individual” is linked to biography, while the Western biomedical approach places the emphasis on the idea of the universal biological individual. However, neither of these points of view use the notion of “the individual” to refer to an identity formed by belonging to a collectivity, which is a conception of the term that has been extensively studied in the field of social sciences.

The anthropologist Duarte [35] studied the notions of “individual” and “person” by comparing the different perceptions of the experience of health and illness among the working classes in modern Brazilian communities and the social segments responsible for biomedical knowledge—as a learned, dominant and official ideology—and found fundamental cultural differences between these two relational models. According to Duarte, referring to Marcel Mauss, a “person” is an entity socially invested with meaning and significance while the modern Western “individual” (thought of as free, autonomous and equal) is “infra-social”, a concrete substrate for the imposition of a social statute.

Duarte shows that the social segments responsible for biomedical knowledge, that is, “individualized” segments, where notions of “person” are manifested, for example, in representations of patients in clinics, hospitals and other public health services, are opposed to representatives of the working class, who tend to perceive diseases in an integrated, relational and holistic way.

Duarte explains this “sociological embarrassment” by the paradox of modern Western societies, which are influenced by the ideology of individualism, whose ideal is the rejection of the universal scheme and consequently the refusal and denial of the totality. He points out that biomedicine has an originary link to some features of individualist ideology as it is connected to universalist/rationalist as well as scientist/physicalist currents of thought. For Duarte, the ideological influence of individualism in biomedicine has increasingly led to a segmentation of domains of knowledge through so-called “medical specialization”; in her opinion, in terms of the techniques and the organization of medical practice, this has an effect similar to that mentioned above because it tends towards the dissolution of the totality of the experience of health and illness ([35], p. 178).

This way of thinking, when applied to the broader discontinuity between the individual and society, shows the need to modify values and realize that models of behaviour are different and vary from culture to culture. Anthropology is a science that can make useful contributions to this discussion because it is defined by its comparative perspective; anthropologists are obliged to relativize the individual in differentiated societies when considering how a person is understood and perceived in his/her society and culture of origin. Psychiatry and psychology, as well as other scientific disciplines are also a “product of a particular culture, at a particular point in time and, thus, an ethnomedical system among others” ([13], p. 222).

According to Duarte [35,57] and Velho [58], the modern Western biomedical culture incessantly attempts to explain signs of sickness by biophysical phenomena and symptomatic behaviour, refusing all other cultural models of explanation, for example those, based on religious and magical beliefs.

This study shows how interviewees find the reasons and justifications for the diagnosis of PTSD within a specific sociocultural context. Although they were attended by a specialized service for victims of violence, the distress reported by them exceeds the trauma that erupted PTSD. Urban violence is certainly one of the elements that makes up their suffering, but not the only element.

According to the 2002 *World Report on Violence and Health*, published by the World Health Organization the impact of collective violence—acts of terrorism, violent crime, wars, genocide, repression and other human rights violations—on mental health is extremely complex and unpredictable, because “it is influenced by a host of factors such as the nature of the conflict, the kind of trauma and distress experienced, the cultural context, and the resources that individuals and communities bring to bear on their situation” ([19], p. 22).

It is important to understand the significance of this document, which presents the issue of violence as relevant to public health, a serious social, economic and health problem in magnitude and scope, which impacts upon society, economy and health, affecting individuals, families and society as a whole.

The interviewees in this research have suffered from the problem of urban violence: however, to individualize this experience completely is to contribute in some way to the naturalization of this phenomenon in society.

## 4. Conclusions

The process of becoming sick, seeking help and being treated should be understood from a cultural and personal perspective. The ethnographic approach, apart from showing individual contexts shrouded in great subjective suffering, also indicates the social suffering experienced in day-to-day life in São Paulo. Social context not only has an impact on the suffering of people requiring medical interventions, but also in the social and political spheres.

This paper argues that it is important for professionals in the field of health, particularly mental health, to think about the question of the individual in the context of the collective. Examining only the victim, without reflecting on the social context, tends to encourage the idea of personal fragility. Limiting the complex problems of violence to the individual sphere does not promote collective mobilization, but can instead contribute to the reduction of social coping mechanisms.

This study suggests some possibilities for intervention by health professionals. Future research should focus on victims of urban violence who do not follow treatment, who do not have access to specialized medical services and the reasons why some patients seek out the health systems and others do not. This approach would give access to cases that go unidentified and do not receive appropriate treatment as pointed by the WHO [19].
